# Impact of the 2022 national formula shortage on clinical decision-making of healthcare providers in switching amino acid formulas for infants with cow’s milk protein allergy: a survey-based study

**DOI:** 10.3389/fped.2024.1328506

**Published:** 2024-03-15

**Authors:** Jessica V. Baran, Jerry M. Brown, Andrew A. Farrar, Lea V. Oliveros, Jesse Beacker, Luke Lamos, Jared Florio, Abigail L. Fabbrini, Michael J. Wilsey

**Affiliations:** ^1^ Office of Medical Education, Florida Atlantic University Charles E. Schmid College of Medicine, Boca Raton, FL, United States; ^2^ Office of Medical Education, Kansas City University College of Osteopathic Medicine, Kansas City, MO, United States; ^3^ Office of Medical Education, Alabama College of Osteopathic Medicine, Dothan, AL, United States; ^4^ Department of Pediatrics, University of South Florida Morsani College of Medicine, Tampa, FL, United States

**Keywords:** amino acid formula, formula shortage, formula recall, infant nutrition, cow’s milk protein allergy, ZSMoments

## Abstract

**Background:**

In 2022, the United States experienced a national shortage of infant formula due to a global supply chain crisis and a large-scale domestic formula recall. The existing literature on healthcare providers' (HCPs) clinical decision-making during formula shortages is limited. This study aims to analyze the factors influencing pediatric HCP clinical decision-making when switching between amino acid formulas (AAF) for managing cow's milk protein allergy (CMPA) in infants under 24 months of age during an unprecedented national formula shortage.

**Methods:**

The study included pediatric HCPs with experience managing CMPA in infants and toddlers under 24 months during the formula shortage from January 2022 to November 2022. A de-identified survey comprising 26 questions examining driving factors used in clinical decision-making was administered to pediatric HCPs using a real-time mobile data collection tool.

**Results:**

Among the surveyed pediatric HCPs (*n* = 75), the factors most frequently considered as “extremely important” when switching to another AAF included safety (85%), tolerability (73%), and efficacy (83%). No statistically significant differences were found in HCP ratings among the listed examined factors of the four AAFs. The availability of specific formulas was the only factor that exhibited a statistically significant difference in perceived performance among pediatric HCPs when comparing the four AAFs (*p* < 0.05).

**Discussion:**

This study elucidates the crucial aspects that influenced pediatric HCPs' selection of AAFs for CMPA management during the 2022 formula shortage. The findings highlight the significance of safety, tolerability, efficacy, and availability in the pediatric HCP decision-making processes.

## 1 Introduction

The United States experienced a shortage of infant formula starting in 2020 due to import restrictions caused by the COVID-19 pandemic (1). This shortage worsened in 2021 due to a global supply chain crisis, reaching a critical peak in February 2022 due to a large-scale product recall from a company supplying 40% of infant formula in the United States (2–4). This shortage significantly impacted the availability of infant formulas across various retail channels, including grocery stores, pharmacies, and online stores (1, 2, 4). Such shortages have profound implications for infants reliant on formula feeding, often leaving parents uncertain about safely feeding their infants (4).

Infant formulas are used for various reasons, including parental choice, breastfeeding supplementation, convenience, or addressing health conditions such as cow's milk protein allergy (CMPA) (1, 5). CMPA, triggered by an immunological reaction to cow's milk protein, affects a significant proportion of children, with symptoms ranging from mild gastrointestinal discomfort to severe anaphylaxis (6–13). Hypoallergenic formulas, such as amino acid formulas (AAF) and extensively hydrolyzed formulas, are the mainstay of CMPA management in formula-fed infants, reducing exposure to cow's milk protein and symptom severity (6, 14). AAFs composed of amino acids and extensively hydrolyzed formulas containing short peptides and amino acids, are well-tolerated in most infants (9, 15–23). However, in cases where infants fail to respond adequately to eHF or exhibit severe allergic reactions, AAFs may be considered as an alternative due to their hypoallergenic nature and ability to meet the nutritional needs of infants with complex medical conditions (23). Factors influencing the choice between these formulas include the severity of CMA, cost, availability, and patient tolerance. Despite being more expensive, AAFs ensure optimal growth while minimizing allergic reactions (18–23).

During the 2022 national formula shortage, pediatric healthcare providers (HCPs) in the United States faced challenges managing CMPA due to the limited availability of hypoallergenic formulas. This study investigated the clinical decision-making process of pediatric HCPs managing infants with CMPA during a national formula shortage crisis. This included an examination of the use of different AAFs as part of CMPA management strategies. Understanding the availability and utilization of various AAFs is crucial for optimizing patient care during formula shortages. Based on the observed increased availability of AAF-1 during the crisis, we hypothesized that HCPs would prioritize the most readily available AAF during the formula shortage and were more likely to continue recommending that formula after the shortage was over.

## 2 Methods

### 2.1 Study objective

This study aims to conduct a cross-sectional analysis of de-identified survey data collected from pediatric HCPs in the United States. The focus is on understanding the key attributes considered by HCPs when switching between AAFs to manage CMPA during a national formula shortage crisis. The study population comprises pediatric HCPs responsible for managing infants with CMPA during the shortage. Through the survey, we aim to assess the clinical decision-making process regarding the utilization of different AAFs and identify factors influencing their choice among pediatric HCPs.

### 2.2 Study design and participants

Inclusion criteria for pediatric HCPs included: greater than 2 years of experience in a clinic-based, pediatric practice setting; specialization in general pediatrics, pediatric gastroenterology, or pediatric allergy/immunology; seeing at least seven newly diagnosed CMPA patients in the past month; managing CMPA in infants aged 0–24 months; and having switched infants' formulas from either AAF-2, AAF-3 or AAF-4 to AAF-1 during the national formula shortage period from January 2022 to November 2022 (Table 1). Exclusion criteria for pediatric HCPs included incomplete data collection. This study received exempt status from the Institutional Review Board (IRB), indicating that it met the criteria for exemption from full IRB review.

**Table 1:** Amino acid-based infant formulas.

Formula group	Trade name	Manufacturer
AAF-1	PurAmino™	Mead Johnson Nutrition, Evansville, IN, USA
AAF-2	Elecare®	Abbott Nutrition, Columbus, OH, USA
AAF-3	Alfamino™	Nestlé Infant Nutrition Inc., Florham Park, NJ, USA
AAF-4	Neocate®	Nutricia, SHS International Ltd., Liverpool, UK

### 2.3 Data collection and variables

The survey was designed to capture pediatric HCP demographic information, formula switch details, availability of the preferred formula, ease of obtaining the new formula, impact of the formula switch on infant health, and the financial burden of the formula switch.

The survey was administered to pediatric HCPs in the United States in English via a mobile-based, data collection application (ZSMoments, by ZS Associates) that allows for the rapid, secure documentation of real-time patient data accurately and freely (19, 20). The survey consisted of 26 questions and took approximately 10–15 min to complete. Survey questions were developed based on a literature review and discussions with pediatric HCPs who manage infants with CMPA. The questionnaire was comprised of yes/no questions and questions rated on a scale from 1 to 10. Scores were categorized into low (1–3), moderate (4–7), and high/extremely (8–10).

### 2.4 Statistical analysis

Descriptive statistics were used to summarize the data, and chi-square tests were conducted to determine the association between demographic variables and formula switch details. Logistic regression analysis was performed to identify factors associated with difficulty obtaining the new formula and increased financial burden. Statistical significance was set at *p* < 0.05. All analyses were carried out using SPSS® analytics software (IBM®, Armon, NY, USA).

## 3 Results

### 3.1 Pediatric HCP demographics

A total of 75 pediatric HCPs were included in data analysis. Pediatric HCP specialties included general pediatrics (64%, *n* = 48), pediatric allergy/immunology (27%, *n* = 20), and pediatric gastroenterology (9%, *n* = 7).

### 3.2 Important formula attributes before vs. after the formula shortage

Among the 75 pediatric HCPs surveyed, the importance of certain factors when choosing an infant formula was assessed (Table 2). The findings indicate that before and after the formula shortages, the pediatric HCPs most frequently rated safety (88% and 93%, respectively) as the most important factor when deciding on a formula for managing CMPA. In addition, before the formula shortage, pediatric HCPs most frequently rated efficacy (87%) and availability (87%) as the tied second-most important factors when deciding on a formula for managing CMPA. However, after the 2022 formula shortage, pediatric HCPs most frequently rated efficacy (92%) as the second most important factor.

**Table 2:** Comparison of perception of importance of attributes among pediatric health care providers.

Factor	Before formula shortage	After formula shortage
Efficacy	87%	92%
Tolerability	81%	89%
Taste/palatability	81%	89%
Provides rapid relief of symptoms	44%	76%*
Safety	88%	93%
Availability	87%	88%
Provides samples	60%	80%
Has different formula preparations available	43%	67%*
Reputable	76%	85%
Used successfully in my practice	76%	81%

* Denotes *p* < 0.05.

### 3.3 Characterization of formula switch to AAF-1

**Figure 1:**
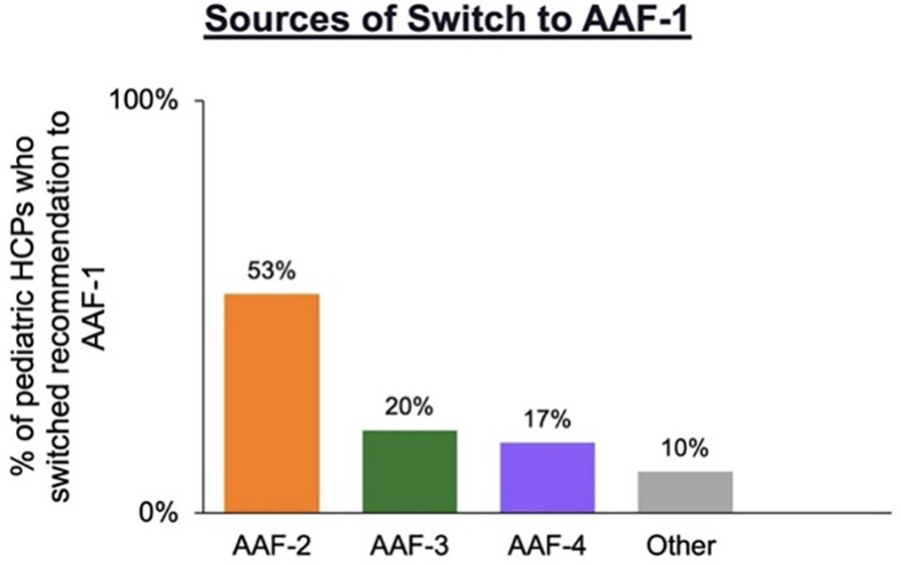
Pediatric HCPs who switched recommendation to AAF-1 (*n* = 75) during the formula shortage by previous AAF recommended.

Of the 75 pediatric HCPs that switched formula to AAF-1 for patients during the shortage, 53% (*n* = 40) were switched from AAF-2, 20% (*n* = 15) from AAF-3, and 17% (*n* = 13) from AAF-4 to (Figure 1).

**Figure 2:**
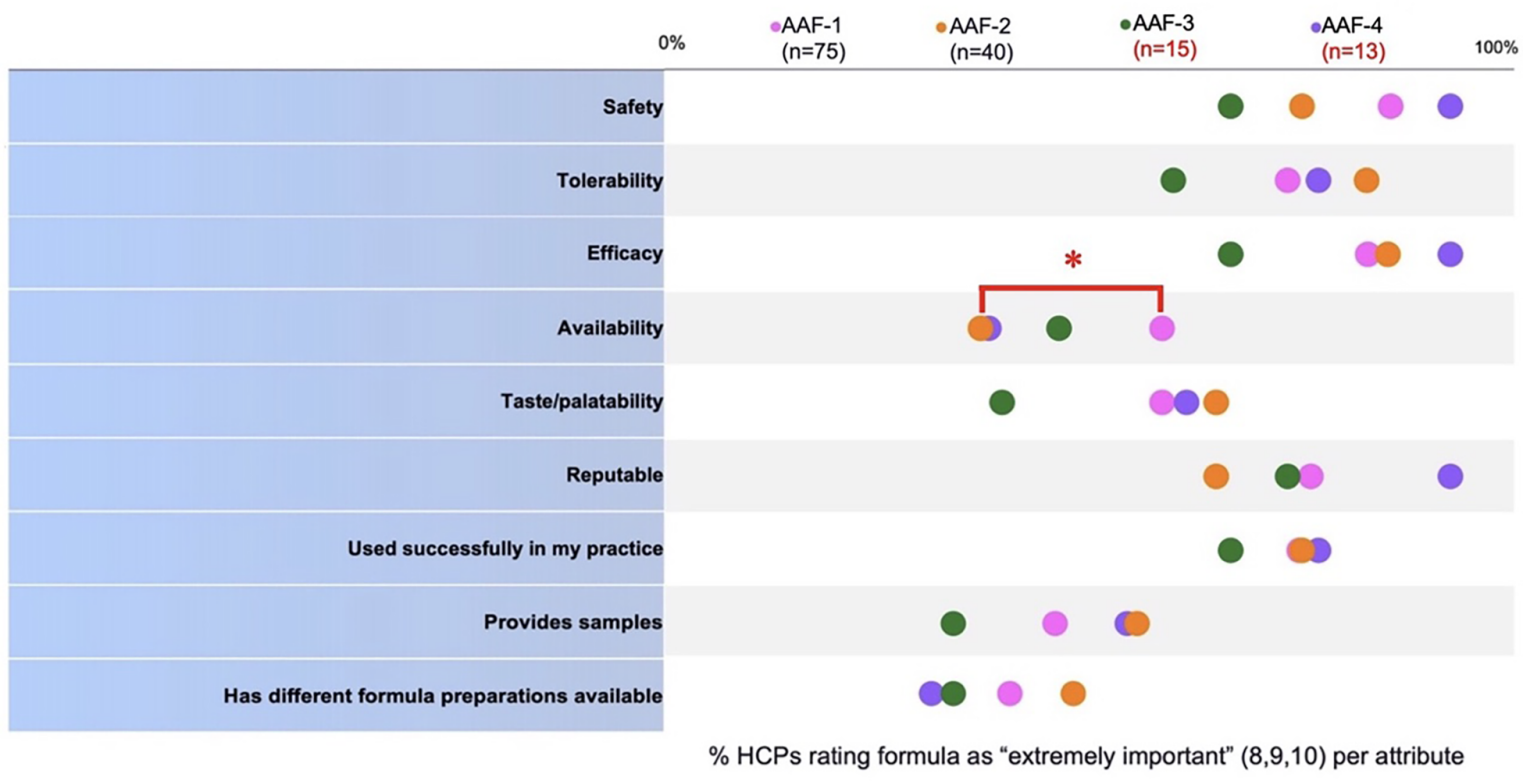
Comparison of pediatric HCP perception of performance of the formulas they used prior to switching (AAF-2, 3, or 4) and pediatric HCP perception of performance of the formula they switched to (AAF-1). *Denotes *p* < 0.05.

When Pediatric HCPs compared quality attributes (Figure 2) of previously recommended formulas to AAF-1 use, AAF-1 was “highly” available significantly more frequently than AAF-2 (*p* < 0.05). No attributes for AAF-1 vs. AAF-3 or vs. AAF-4 were rated as performing significantly better. The percentage of pediatric HCPs that rated each attribute as “extremely important” is listed in Supplementary Table S1.

### 3.4 Quality perception of AAF-1 and future preference after switch

**Figure 3:**
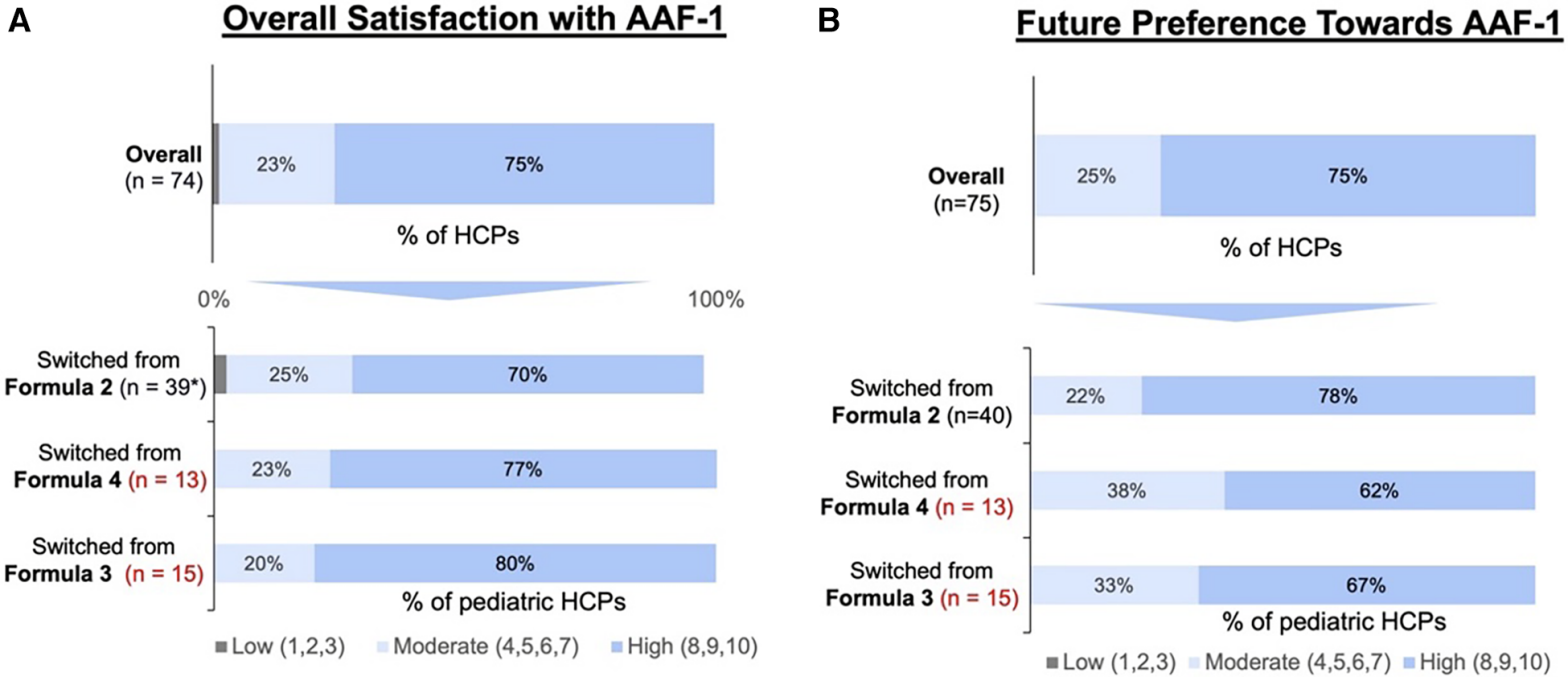
Pediatric HCP rating for: (**A**) AAF-1 overall quality perception after switching formulas during the shortage and (**B**) AAF-1 future preference after resolution of the shortage. (*Forty pediatric HCPs initially recommended AAF-2 and then switched to AAF-1. However, only 39 of the 40 pediatric HCP's felt as if they has enough experience with AAF-1 to rate their satisfaction. Therefore, only 74 pediatric HCPs were included in the overall sample).

Pediatric HCP satisfaction ratings (Figure 3A) and future preference after switching to AAF-1 (Figure 3B) were collected. Of 98% (*n* = 39) pediatric HCPs who switched from AAF-2, 70% (*n* = 28) were “highly” satisfied with AAF-1% and 78% (*n* = 31) had a “high” future preference to AAF-1. Of 13 pediatric HCPs that switch from initially recommended AAF-4, 77% (*n* = 10) were “highly” satisfied with AAF-1 (Figure 3) and 62% (*n* = 9) had a “high” future preference to AAF-1. Of 15) who switched from AAF-3, 80% were “highly” satisfied with AAF-1 (Figure 3) and 67% (*n* = 10) rated “high” future preference.

### 3.5 HCP perception of AAF-1 benefits

**Figure 4:**
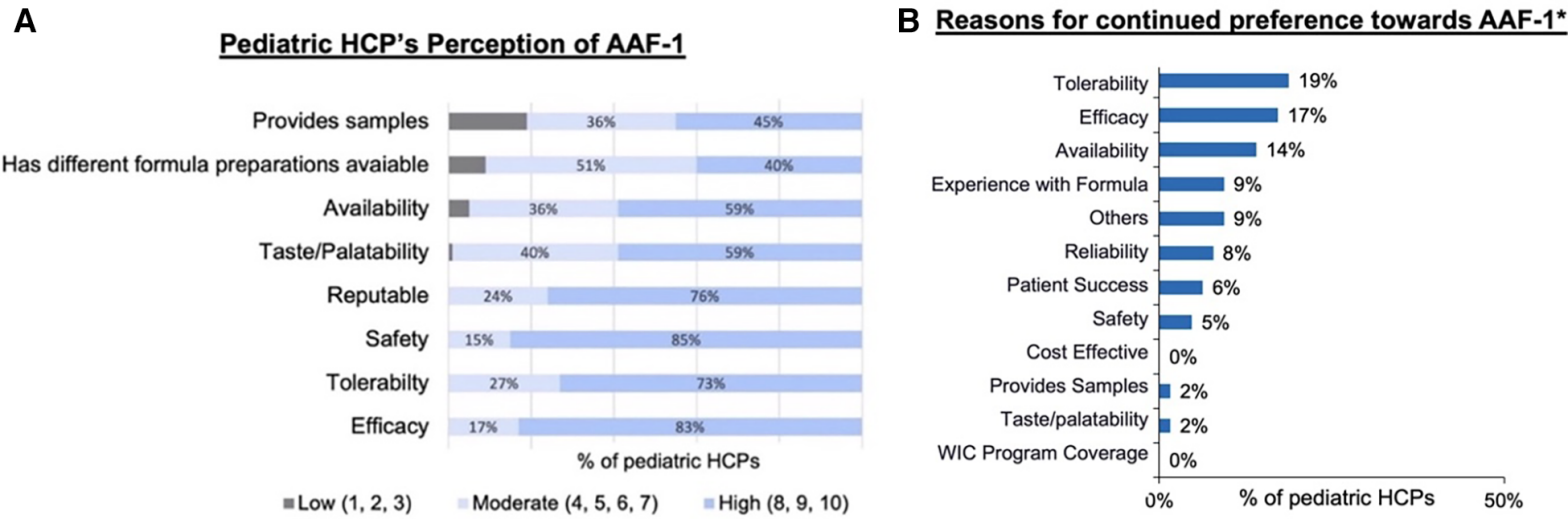
(**A**) Pediatric HCP rating of perception of AAF-1 performance. (**B**) Reason pediatric HCPs who rated these attributes as extremely important continued preference towards AAF-1. *Includes pediatric HCPs that rated these attributes at extremely important (8–10).

Specific beliefs and benefits of AAF-1 were rated by pediatric HCPs who switched to AAF-1 during the shortage (Figure 4A). Of the 75 pediatric HCPs included in the study overall, 75% (*n* = 56) would continue to recommend AAF-1 following resolution of the shortage. AAF-1 was reported as having “high” tolerability in 73% (*n* = 55) and 59% (*n* = 44) considered AAF-1 to have “high” taste/palatability. In addition, 83% (*n* = 62) considered AAF-1 to have “high” efficacy and 85% (*n* = 64) considered AAF-1 to have “high” safety. Figure 4B depicts the attributes pediatric HCPs rated for continued preference of AAF-1.

## 4 Discussion

In this cross-sectional analysis, we evaluated the driving factors utilized by pediatric HCPs in clinical decision making when switching between AAFs to manage CMPA in infants ≤24 months before and after the 2022 formula shortage. Here, we report a cohort of 75 pediatric HCPs that switched their formula recommendation from AAF-2-4 to AAF-1 during the shortage.

Understanding what influences clinical decision-making among pediatric HCPs managing infants with CMPA is crucial. Once a diagnosis is established, pediatric HCPs need to develop an individualized treatment plan for infants based on symptom severity and available resources. Clinical decision-making involved evaluating various management options, considering patient specific needs and circumstances, and make informed decisions about the most suitable management strategies. This process can optimize the effectiveness and safety of management strategies in pediatric patients. Understanding clinical decision making is especially important in times of formula shortage. This understanding allows pediatric HCPs to be better prepared for management dilemmas, more aware of the various treatment options and their respective benefits, and overall, more successful in managing infants with CMPA using hypoallergenic formulas.

Considering the formula shortage crisis, it's crucial to examine how different amino acid formulas (AAF) were affected and their implications for managing infants with cow's milk protein allergy (CMPA). The shortage significantly disrupted the supply chain, particularly impacting the availability of AAFs, which are essential for infants with severe CMPA. This scarcity emphasized the importance of understanding the availability and accessibility of various AAFs during crises. It also highlighted the need for proactive measures to ensure an adequate supply and distribution of specialized formulas to mitigate potential risks associated with shortages. Collaborative efforts among stakeholders are essential to address these challenges and develop strategies to safeguard the availability of essential formulas for vulnerable populations during crises (24, 25).

Our findings from this survey indicate that pediatric HCPs during the national formula crisis in the US rated availability significantly higher when comparing AAF-1 to AAF-3, with availability driving most switches to AAF-1 during the shortage. Furthermore, post-shortage, most pediatric HCPs expressed intent to continue recommending AAF-1, highlighting its sustained preference.

Safety remained the most important factor when choosing a formula both before and after the formula shortage. Interestingly, availability shifted from being tied-second most important before the shortage to efficacy becoming second most important after the shortage. During the formula shortage, the increased availability of AAF-1 overshadowed other formula attributes, significantly impacting clinical decision-making among pediatric HCPs. This insight into decision-making during and after formula shortages can inform improvements in CMPA patient treatment and management.

After shifting to AAF1, most pediatricians expressed high satisfaction with AAF-1 and demonstrated a strong future preference for AAF-1. This finding underscores the importance of considering pediatricians' satisfaction and preferences when evaluating the effectiveness and acceptability of hypoallergenic formulas. Future studies should delve deeper into the reasons behind pediatricians' satisfaction with AAF-1 and explore potential factors influencing formula preference.

Limitations include the study's focus on specific pediatric HCPs in the United States, potentially limiting generalizability (19). These findings may not be applicable to HCPs in other countries, potentially limiting the generalizability of the results. In addition, the data collected from pediatric HCPs were based on self-reporting through a survey. This method relies on participants' recall and subjective responses, which can introduce biases such as recall bias or social desirability bias. The accuracy and reliability of the data depend on the participant's ability to recall information. Further, this study utilizes a cross-sectional design, which captures data at a specific point in time, limiting the ability to establish causal relationships or assess changes over time. Future research should explore potential confounding variables like symptom severity or socioeconomic factors influencing pediatric HCP perceptions.

## 5 Conclusion

Our study, the first survey of its kind, highlights the impact of formula availability on clinical decision-making among pediatric HCPs managing CMPA infants during a national formula shortage. AAF-1’s increased availability drove most switches, underscoring its importance amidst formula shortages. However, our findings suggest that safety, tolerability, and efficacy also influenced decision-making, albeit to a lesser extent than availability. This emphasizes the multifaceted nature of pediatric HCP decision-making. Further research is warranted to validate these findings.

## Data Availability

The raw data supporting the conclusions of this article will be made available by the authors, without undue reservation.

## References

[1] Abrams SA Duggan CP. Infant and child formula shortages: now is the time to prevent recurrences. Am J Clin Nutr. (2022) 116:289–92. 10.1093/ajcn/nqac149 35580593 PMC9348970

[2] Kalaitzandonakes M Ellison B Coppess J. Coping with the 2022 infant formula shortage. Prev Med Rep (2023) 32:102123. 10.1016/j.pmedr.2023.102123 36798794 PMC9926015

[3] Asiodu IV. Infant formula shortage: this should not be our reality. J Perinat Neonatal Nurs. (2022) 36:340–3. 10.1097/JPN.0000000000000690 36288439 PMC9623470

[4] Jung J Widmar NO Ellison B. The curious case of baby formula in the United States in 2022: cries for urgent action months after silence in the midst of alarm bells. Food Ethics. (2023) 8:4. 10.1007/s41055-022-00115-1 36533216 PMC9734447

[5] O’Connor NR. Infant formula. Am Fam Physician. (2009) 79:565–70. 19378873

[6] Vandenplas Y Brough HA Fiocchi A Miqdady M Munasir Z Salvatore S Current guidelines and future strategies for the management of cow’s milk allergy. J Asthma Allergy. (2021) 14:1243–56. 10.2147/JAA.S276992 34712052 PMC8548055

[7] Vitaliti G Cimino C Coco A Pratico AD Lionetti E. The immunopathogenesis of cow’s milk protein allergy (CMPA). Ital J Pediatr. (2012) 38:35. 10.1186/1824-7288-38-71 22824011 PMC3441837

[8] Flom JD Sicherer SH. Epidemiology of cow’s milk allergy. Nutrients. (2019) 11. 10.3390/nu11051051 31083388 PMC6566637

[9] Meyer R Groetch M Venter C. When should infants with cow’s milk protein allergy use an amino acid formula? A practical guide. J Allergy Clin Immunol Pract. (2018) 6:383–99. 10.1016/j.jaip.2017.09.003 29109046

[10] Lozinsky AC Meyer R Anagnostou K Dziubak R Reeve K Godwin H Cow’s milk protein allergy from diagnosis to management: a very different journey for general practitioners and parents. Children (Basel). (2015) 2:317–29. 10.3390/children2030317 27417366 PMC4928770

[11] Caffarelli C Baldi F Bendandi B Calzone L Marani M Pasquinelli P Cow’s milk protein allergy in children: a practical guide. Ital J Pediatr. (2010) 36:5. 10.1186/1824-7288-36-5 20205781 PMC2823764

[12] Walsh J O’Flynn N. Diagnosis and assessment of food allergy in children and young people in primary care and community settings: NICE clinical guideline. Br J Gen Pract. (2011) 61:473–5. 10.3399/bjgp11X583498 21722479 PMC3123497

[13] Vandenplas Y De Greef E Devreker T. Treatment of cow’s milk protein allergy. Pediatr Gastroenterol Hepatol Nutr. (2014) 17:1–5. 10.5223/pghn.2014.17.1.1 24749081 PMC3990777

[14] Koletzko S Niggemann B Arato A Dias JA Heuschkel R Husby S Diagnostic approach and management of cow’s-milk protein allergy in infants and children: ESPGHAN GI committee practical guidelines. J Pediatr Gastroenterol Nutr. (2012) 55:221–9. 10.1097/MPG.0b013e31825c9482 22569527

[15] Host A Koletzko B Dreborg S Muraro A Wahn U Aggett P Dietary products used in infants for treatment and prevention of food allergy. Joint statement of the European society for paediatric allergology and clinical immunology (ESPACI) committee on hypoallergenic formulas and the European society for paediatric gastroenterology, hepatology and nutrition (ESPGHAN) committee on nutrition. Arch Dis Child. (1999) 81:80–4. 10.1136/adc.81.1.80 10373144 PMC1717972

[16] Beacker J Brown JM Florio J Baran JV Lamos L Oliveros L Clinician experience with using hypoallergenic formulas to treat infants with suspected cow’s milk protein allergy: a secondary analysis of a prospective survey cohort. Pediatr Gastroenterol Hepatol Nutr. (2023) 26(5):277–83. 10.5223/pghn.2023.26.5.277 37736218 PMC10509022

[17] Fabbrini AL Farrar AA Brown JM Oliveros LV Florio J Beacker J Navigating formula shortages: associations of parental perspectives on transitioning to alternative infant formulas for cow’s milk protein allergy during the 2022 national formula shortage. Front Allergy. (2024) 4:1333570. 10.3389/falgy.2023.1333570 38260176 PMC10801258

[18] Ludman S Shah N Fox AT. Managing cows’ milk allergy in children. Br Med J. (2013) 347:f5424. 10.1136/bmj.f5424 24041704

[19] Wilsey MJ Florio J Beacker J Lamos L Baran JV Oliveros L Extensively hydrolyzed formula improves allergic symptoms in the short term in infants with suspected cow’s milk protein allergy. Nutrients. (2023) 15. 10.3390/nu15071677 37049517 PMC10096968

[20] Wilsey MJ Baran JV Lamos L Beacker J Florio J Oliveros L Short-term symptom improvement in infants with suspected cow’s milk protein allergy using amino acid formula: a prospective cohort analysis. Front Nutr*.* (2023) 10:1208334. 10.3389/fnut.2023.1208334 37408987 PMC10318537

[21] Meyer R Smith C Sealy L Mancell S Marino LV. The use of extensively hydrolysed and amino acid feeds beyond cow’s milk allergy: a national survey. J Hum Nutr Diet. (2021) 34:13–23. 10.1111/jhn.12794 32820586

[22] Nowak-Wegrzyn A Czerkies LA Collins B Saavedra JM. Evaluation of hypoallergenicity of a new, amino acid-based formula. Clin Pediatr (Phila). (2015) 54:264–72. 10.1177/0009922814557785 25395609

[23] D'Auria E Salvatore S Acunzo M Peroni D Pendezza E Di Profio E Hydrolysed formulas in the management of cow’s milk allergy: new insights, pitfalls and tips. Nutrients. (2021) 13(8):2762. 10.3390/nu13082762 34444922 PMC8401609

[24] Doherty T Coutsoudis A McCoy D Lake L Pereira-Kotze C Goldhagen J Is the US infant formula shortage an avoidable crisis? Lancet. (2022) 400(10346):83–4. 10.1016/S0140-6736(22)00984-9 35654081

[25] Mulherin DW Kumpf V Shingleton K. Managing nutrition support product shortages: what have we learned? Nutr Clin Pract. (2023) 38(1):27–45. 10.1002/ncp.10927 36309480

