# Supplementary material for: Impact of the 2022 national formula shortage on clinical decision-making of healthcare providers in switching amino acid formulas for infants with cow’s milk protein allergy: a survey-based study
Source: Front Pediatr. 2024 Mar 15;12:1328506. doi: 10.3389/fped.2024.1328506 (PMC10978596; doi:10.3389/fped.2024.1328506)
Supplement: Supplementary file 1 [file Table1.docx]

|  | **AAF-1** | **AAF-2** | **AAF-3** | **AAF-4** |
| --- | --- | --- | --- | --- |
| Safety | 85% | 75% | 67% | 92% |
| Tolerability | 73% | 83% | 60% | 77% |
| Efficacy | 83% | 85% | 67% | 92% |
| Availability | 59% | 38% | 47%* | 38% |
| Taste/palatability | 59% | 65% | 40% | 62% |
| Reputation | 76% | 65% | 73% | 92% |
| Used successfully in my practice | 75% | 75% | 67% | 77% |
| Provides samples | 45% | 55% | 33% | 54% |
| Has different formula preparations available | 40% | 48% | 33% | 31% |

**Supplementary Table 1.** Comparison of pediatric HCP perception of performance of the formulas they used prior to switching (AAF-2, 3, or 4) and pediatric HCP perception of performance of the formula they switched to (AAF-1). *Denotes p<0.05.
